# Necrosis in Preoperative Cross-Sectional Imaging and Postoperative Histology Is a Diagnostic Marker for Malignancy of Adrenocortical Tumors

**DOI:** 10.3390/curroncol32010025

**Published:** 2025-01-01

**Authors:** Agata Dukaczewska, Stephan R. Marticorena Garcia, Simon Ponsel, Alexandra Webster, Frederike Butz, Eva M. Dobrindt, Johann Pratschke, Peter E. Goretzki, David Horst, Martina T. Mogl, Catarina A. Kunze

**Affiliations:** 1 Department of Surgery, Campus Charité Mitte and Campus Virchow-Klinikum, Charité–Universitätsmedizin Berlin, Corporate Member of Freie Universität Berlin, Humboldt-Universität zu Berlin and Berlin Institute of Health, Charitéplatz 1, 10117 Berlin, Germany; agata.dukaczewska@charite.de (A.D.); frederike.butz@charite.de (F.B.); eva-maria.dobrindt@charite.de (E.M.D.); johann.pratschke@charite.de (J.P.); pgoretzki@icloud.com (P.E.G.); 2 Department of Radiology, Charité–Universitätsmedizin Berlin, Corporate Member of Freie Universität Berlin, Humboldt-Universität zu Berlin and Berlin Institute of Health, Charitéplatz 1, 10117 Berlin, Germany; stephan.marticorena-garcia@charite.de (S.R.M.G.); simonponsel@gmail.com (S.P.); alexandra.webster@charite.de (A.W.); 3 Institute of Pathology, Charité–Universitätsmedizin Berlin, Corporate Member of Freie Universität Berlin, Humboldt-Universität zu Berlin and Berlin Institute of Health, Charitéplatz 1, 10117 Berlin, Germany; david.horst@charite.de (D.H.); catarina.kunze@charite.de (C.A.K.); 4 German Cancer Consortium (DKTK), Partner Site Berlin and German Cancer Research Center (DKFZ), Im Neuenheimer Feld 280, 69120 Heidelberg, Germany

**Keywords:** adrenocortical carcinoma, adrenocortical adenoma, adrenalectomy, tumor necrosis, computed tomography, magnetic resonance imaging

## Abstract

Necrosis in postoperative histology has been reported as being specific for adrenocortical carcinoma (ACC) compared to adenoma. We therefore retrospectively analyzed the diagnostic accuracy of the finding of necrosis in preoperative cross-sectional imaging and postoperative histology as a marker for ACC in our patient cohort. Among the 411 adrenalectomies in 396 patients performed between 2008 and April 2022, 30 cases of ACC (7.6%) were identified, with one tumor measuring less than 40 mm excluded. All 45 benign adrenocortical tumors of at least 40 mm in diameter, including Cushing, Conn, and hormonally inactive adenomas, served as controls. Preoperative imaging was available for 40 benign and 27 malignant adrenocortical tumors. In total, 10 of 40 (25%) benign adrenocortical tumors and 22 of 27 (81%) ACCs showed signs of possible necrosis in preoperative imaging. Pathologic examination confirmed necrosis in 1 of 40 (2.5%) benign tumors and in 26 out of 27 (96%) malignant tumors. The specificities of possible necrosis in preoperative imaging and necrosis in histology for diagnosing ACC were 75% and 97.5%, respectively, whereas the sensitivities were 81% and 96%, respectively. Signs of possible necrosis in radiologic imaging and tumor necrosis in histology proved to be very good predictive markers for the diagnosis of malignant adrenocortical tumors.

## 1. Introduction

Adrenocortical cancer (ACC) is a rare disease that is diagnosed in less than 2% of accidentally detected adrenal tumors [1]. Owing to increasing numbers of cross-sectional imaging examinations, a growing number of incidentalomas have been observed, accounting for 2.3% of all computed tomography scans [2]. Given the poor prognosis of ACC [3], it is important to distinguish malignant adrenal tumors from the multitude of incidentalomas, to offer prompt surgical and medical treatment to the affected patients while avoiding overtreatment for benign tumors. Currently, preoperative histological confirmation of adrenal tumors is lacking because of the high risk of tumor dissemination during biopsies [4,5]. Thus, careful noninvasive evaluation of incidentalomas is needed. Computed tomography (CT) and magnetic resonance imaging (MRI) are noninvasive preoperative imaging methods. However, quantitative analysis is limited to the evaluation of fatty tissue content in adrenal tumors as well as the tumor size [4,5].

The need to correlate the pathologic and radiologic findings to enhance early diagnosis in malignant tumors has repeatedly been underscored [6,7,8]. The histopathological distinction between adrenocortical adenoma and carcinoma relies primarily on various multifactorial scoring systems, such as the Weiss system [9], the Helsinki score [10], and the reticulin algorithm [11]. However, according to the World Health Organization, no single system has been proven to be entirely sensitive or specific [12]. Nevertheless, a common feature among these systems is the presence of necrosis, which serves as an indicator of malignancy. Recently, confluencing necrosis in tumor histology has been highlighted as an easily measurable and reliable single parameter that is predictive of malignancy in adrenocortical tumors [13]. In addition to the infiltration of the tumor capsule and invasion of vessels in large, advanced adrenocortical carcinomas, necrosis stands out as one of the few histologic findings detectable in CT and MRI scans. Thus, we hypothesize that preoperative changes associated with tumor necrosis detected in CT and MRI can predict tumor malignancy.

The purpose of this study was to analyze the diagnostic accuracy of tumor necrosis by preoperative CT/MRI and postoperative histopathology as a marker for malignancy of adrenocortical tumors.

## 2. Materials and Methods

### 2.1. Patient Demographic and Clinical Data

**Figure 1:**
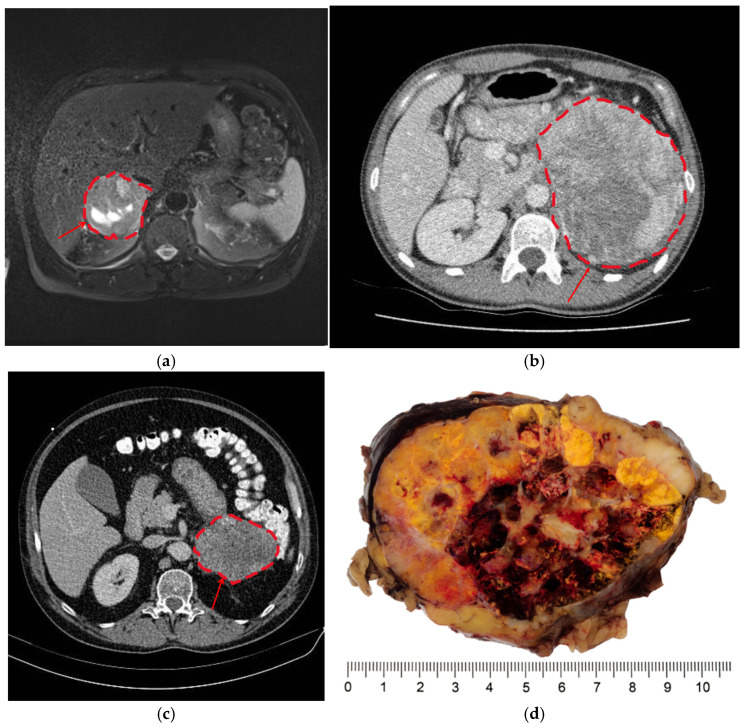
(**a**) A T2-weighted magnetic resonance image of a 59-year-old male patient with a 17-OH-progesterone-producing right-sided ACC (arrow), measuring 85 mm in the largest diameter. Hyperintense lesions correspond with necrosis; (**b**) contrast-medium-enhanced computed tomography in a 25-year-old patient with a cortisol- and androstendion-producing left-sided ACC measuring 150 mm, showing diffuse areas of necrosis (arrow); (**c**) contrast-enhanced computed tomography in a 49-year-old male with a cortisol- and 17-OH-progesterone-producing left-sided ACC (arrow), measuring 130 mm in the largest diameter, with inhomogeneous enhancement, calcifications, and hypodense areas suspicious of necrosis; (**d**) a picture of the excised ACC after formalin fixation in correlation with the preoperative computed tomography scan (Figure 1c). The large tumor shows indistinct borders and a variegated cut surface with central hemorrhage and necrotic areas.

All the adrenalectomies performed in our clinic from January 2008 to April 2022 included 411 adrenalectomies in 396 patients. Among all the excised tumors, 30 were diagnosed as ACC. As adrenocortical tumors measuring less than 40 mm are extremely rarely malignant [14], only tumors measuring at least 40 mm were included. Furthermore, all pheochromocytomas were excluded, as the preoperative diagnosis can be confirmed biochemically. Sarcomas, lymphomas, hemangiomas, bilateral adrenal hyperplasia and tumor recurrence, adrenal metastases, oncocytomas, fibromas, schwannomas, and all preoperatively diagnosed myelolipomas and adrenal cysts were ruled out. Native and contrast-medium-enhanced CT and MRI scans were used as index tests. Ex vivo histopathology served as a reference. All images were analyzed by two radiologists (S.P., with 5 years of experience in clinical radiology, supervised by a consultant radiologist, S.R.M.G., with more than 10 years of experience in clinical radiology). Both examiners were blinded to the final diagnosis. Intratumoral necrosis was defined as hyperintense lesions in fat-saturated/non-fat-saturated T2-weighted images with corresponding hypointensity in T1-weighted images on MRI [15] or nonenhancing and low-attenuation lesions on CT [16] (Figure 1).

### 2.2. Histological Evaluation

**Figure 2:**
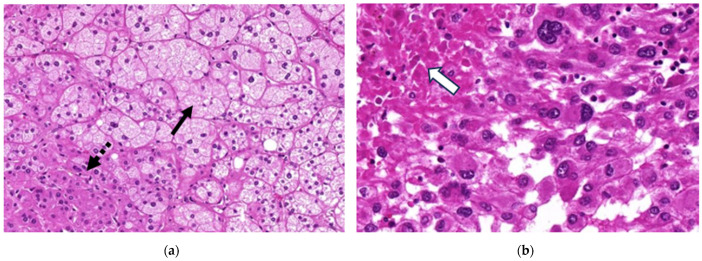
(**a**) Microscopic image of an adrenocortical adenoma (a 56-year-old female patient with hyperaldosteronism) showing nests of lipid-rich cells (upper right corner, continuous arrow) and compact lipid-poor cells (lower left corner, dotted arrow) with small and uniform nuclei. Necrosis, increased mitotic activity, or nuclear pleomorphism are absent (H&E stain, 100 × magnification). (**b**) In contrast, the ACC (a 78-year-old female patient with a hormonally inactive tumor) is composed of solid to diffuse growing tumor cells with large highly atypical nuclei and frequent confluencing necrosis (upper left corner, block arrow; H&E stain, 200 × magnification).

For all patients, diagnostic hematoxylin and eosin (H&E)-stained slides were obtained at the Institute of Pathology, Charité–Universitätsmedizin Berlin. Histological samples of ACCs and benign adrenocortical tumors were reevaluated by an experienced pathologist (C.A.K., with more than 6 years of experience in clinical pathology, specialized in endocrine tumors) for the diagnosis and the presence of tumor necrosis (Figure 2).

### 2.3. Statistical Analysis

Continuous variables are displayed as medians (range), and categorical variables are displayed as frequencies (percentages). As the patient cohorts were small and not normally distributed, as determined by the Shapiro–Wilk normality test, a two-sided nonparametric bootstrap t test with pool sampling was used for group comparisons of variables [17]. This test addresses issues of the dataset appropriately to estimate *p* values most accurately compared with standard *t* test, proportion tests, or other bootstrapping methods. For extremely small group sizes, pooled bootstrapping is not reliable and hence is not computable. This applied to aldosterone-producing tumors and tumors that simultaneously produce cortisol and steroid hormones in our study. As a rough estimation for these two variables, a two-sided test of equal or given proportions (Prop.Test) was used for group comparisons. Because of extremely small group sizes and nonnormality, they cannot be interpreted reliably. Owing to the lack of a normal distribution, the results of the two-sided Prop.Test must be taken with caution. A receiver operating characteristic (ROC) analysis was performed to evaluate the accuracy of the regression prediction of malignancy using tumor size. For the ROC curve, the dataset was split into 80% training data and 20% testing data. A multivariate logistic regression model was applied to assess the correlations between patient sex and age, tumor size, tumor side, hormonal activity, and the occurrence of areas suspected of necrosis with malignancy. A correction of *p* values for multiple comparisons was conducted using the Bonferroni–Holm method. The significance level was set to 0.05. Statistical analyses were performed using “R” statistical software (basic package, npboottprm package, version 2023 09.0+463).

## 3. Results

**Figure 3:**
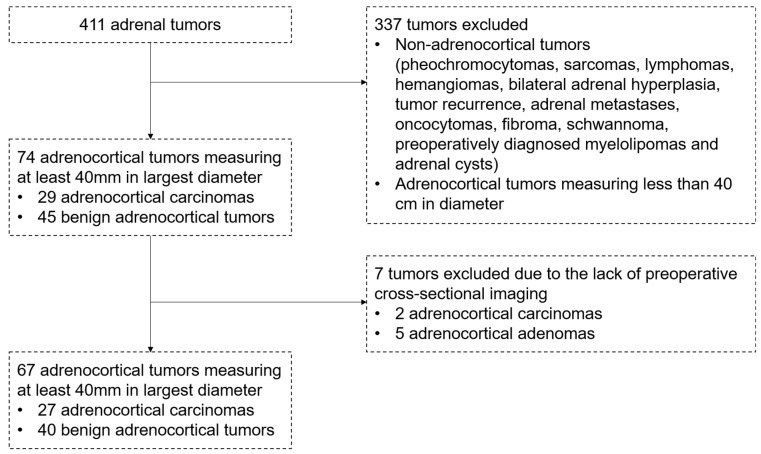
Flowchart depicting the inclusion/exclusion criteria for adrenocortical tumors.

Out of the 30 ACCs, 29 met the inclusion criteria; one ACC of 29 mm in diameter, which was diagnosed in a patient with Li–Fraumeni syndrome, was excluded from the analysis. All 45 benign adrenocortical tumors of at least 40 mm in diameter were chosen as controls. For 5 patients with benign tumors and 2 patients with malignant tumors, no cross-sectional imaging was available retrospectively. Thus, 27 ACCs and 40 benign tumors, including Conn (*n* = 2) and Cushing adenomas (*n* = 20), adenomas without hormonal activity (*n* = 18), and 27 malignant tumors were included in the study. A schematic illustration of the inclusion of adrenal tumors in our study is depicted in Figure 3. The characteristics of the final study cohort are presented in Table 1.

An overview of the available preoperative cross-sectional imaging for benign and malignant adrenocortical tumors in our patient cohort is summarized in Table 2. The median time between the last cross-sectional imaging and the operation was 51.5 (range 1–261) days for all tumors, and 68.5 (range 1–261) days for benign and 23.5 (range 1–212) days for malignant adrenocortical tumors.

In histology, necrosis was confirmed in 26 out of 27 ACCs (96%) and in one benign tumor (2.5%). Necrosis was identified in 23 out of 27 (81%) malignant tumors and in 10 out of 40 (25%) benign tumors by preoperative cross-sectional in vivo CT/MRI.

For the identification of malignancy by necrosis, a sensitivity and specificity of 81%/75% were calculated for in vivo cross-sectional imaging, and a sensitivity and specificity of 96%/97% were calculated for ex vivo histopathology. Tumor necrosis, evaluated as a single marker of malignancy in preoperative cross-sectional imaging, demonstrated a positive predictive value (PPV) of 69%, a negative predictive value (NPV) of 86%, and an overall accuracy of 78%. In histology, it achieved a PPV of 96%, NPV of 97.5%, and an accuracy of 97%.

**Figure 4:**
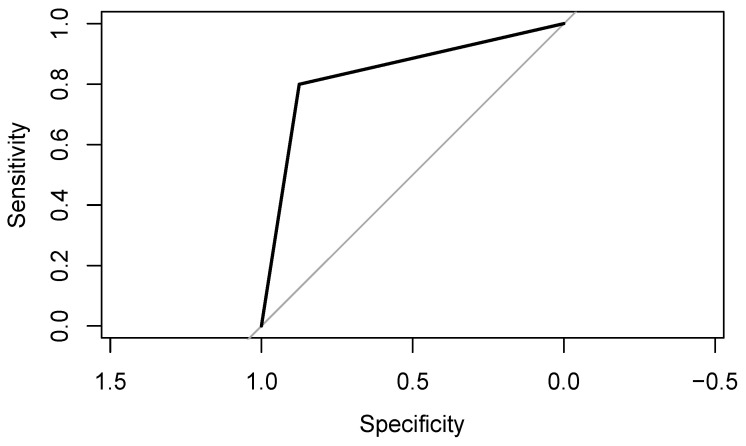
Receiver operating characteristic (ROC) curve representing sensitivity versus specificity of logistic regression using only the size of adrenocortical tumors as an explanatory variable.

The median volume of the tumors in imaging was 63.901 cm^3^ for benign and 378.288 cm^3^ for malignant tumors. In histology, the median diameters of the excised tumors were 50 mm (interquartile range (IQR) 45–60 mm) and 100 mm (IQR 76–137.5 mm), respectively. Thus, the ACCs were significantly larger than the adrenocortical adenomas in our patient cohort (*p* < 0.001), with a sensitivity and specificity of 80%/90% for identifying malignant tumors in the ROC analysis (area under the receiver operating characteristic curve (AUROC) of 0.8735) (Figure 4).

The linear correlation between the presence of confluent necrosis in histology and the malignancy of adrenocortical tumors was nearly perfect (*p* < 0.001). Thus, to avoid a multicollinearity, the presence of confluent necrosis in histology was excluded from the regression and was regarded separately. As both the tumor volume in cross-sectional imaging and the tumor diameter in histology represent the size of the tumor, logistic regression was performed separately for both parameters. The tumor diameter in histology had greater explanatory power, as it had a lower *p* value (0.00169 vs. 0.00654) and led to a lower Akaike information criterion (AIC) (49.266 vs. 58.084). Sex, age, tumor diameter, and overall hormonal activity had no significant influence on tumor malignancy, as shown in Table 3. However, there was a strong relationship between both the tumor diameter (*p* = 0.002) and the presence of areas suspected of necrosis in cross-sectional imaging (*p* = 0.004) and the postoperative diagnosis of ACC (Table 3).

The overproduction of steroid hormones, including androgens and 17-OH-progesterone, but not of cortisol, was significantly associated with tumor malignancy in our patient cohort. The results of the endocrinologic work-up of hormonally active tumors in our patient cohort are summarized in Table 4.

## 4. Discussion

In the present study, we showed that necrosis is an independent predictive biomarker of malignant adrenocortical tumors, which was confirmed by in vivo preoperative CT/MRI imaging and ex vivo histopathology with high diagnostic accuracy. Our results indicate that identifying necrosis in cross-sectional scans facilitates the selection of patients with adrenocortical tumors requiring a prompt surgical intervention while helping to prevent the overtreatment of benign tumors. Furthermore, in line with the previous results [13], the detection of confluent necrosis enormously simplifies the pathological evaluation of histological samples of adrenocortical masses in terms of their malignant potential.

Necrosis arises from metabolic stress caused by depletion of the vascular supply in rapidly growing tumors. Additionally, immune cells appear to worsen tumor necrosis through the mechanism of ferroptosis [18]. It is linked to poor prognosis in various cancers, including soft tissue sarcomas [19], non-small-cell lung cancer [20], breast cancer [21], hepatocellular carcinoma [22], and renal cell carcinoma [23]. In adrenocortical tumors, necrosis is utilized in traditional scoring systems by Weiss [9] and van Slooten [24] to differentiate malignancy. The simplified Helsinki score highlights necrosis as the most effective predictor of malignancy [10]. The presence of necrosis can simply be evaluated in a basic hematoxylin and eosin staining of a histological sample, with a high sensitivity and specificity [13]. Hematoxylin and eosin staining is widely available, also in low-resource settings where access to advanced immunohistological techniques may be limited. It also enables the detection of further markers of malignancy, such as capsular and vascular invasion, high mitotic rates, nuclear hyperchromasia, nuclear atypia, and calcifications [9,13,24]. However, immunohistological evaluation of the proliferation index further enhances specificity in assessing the malignant potential of adrenocortical tumors [10]. Combining these approaches allows for a more accurate and nuanced diagnostic evaluation. Furthermore, molecular profiling has emerged as an additional tool for understanding ACC [25,26]. The integration of molecular data, including gene expression, methylation, and chromosomal alterations, with clinical and histopathological parameters can significantly improve prognostic accuracy, identifying patients with favorable, good, intermediate, and poor prognoses to guide adjuvant therapy decisions [25]. While molecular profiling is not yet routine in clinical practice, advancements in analyzing formalin-fixed, paraffin-embedded tumor samples suggest its potential for widespread implementation, laying the foundation for precision medicine and novel therapeutic targets in ACC [25,26].

Necrosis can be easily detected in preoperative cross-sectional imaging. The assessment of necrosis in cross-sectional imaging was previously proposed by Yalon et al. and Garay-Lechuga et al., who reported sensitivities of 76% and 100%, respectively, and specificities of 99.4% and 97.14%, respectively, in detecting adrenocortical carcinoma [16,27]. In line with the findings of Garay-Lechuga et al., this study investigated necrosis as a marker for malignancy of adrenocortical tumors in both preoperative cross-sectional imaging and postoperative histology within a single-center patient cohort. In the present study, the identification of necrosis in cross-sectional imaging yielded a better sensitivity of 81%, but a lower specificity of 75%, compared to the findings of Yalon et al., and both lower sensitivity and lower specificity compared to the findings of Garay-Lechuga et al. The differences in the diagnostic accuracy of preoperative cross-sectional imaging between these studies may be attributed to the heterogeneity of examination protocols of retrospectively available radiologic imaging in all three studies. Thus, further investigations, preferably prospective, are needed to evaluate the finding of necrosis as a marker predictive of malignancy in adrenocortical tumors via standardized examination protocols in cross-sectional imaging.

**Figure 5:**
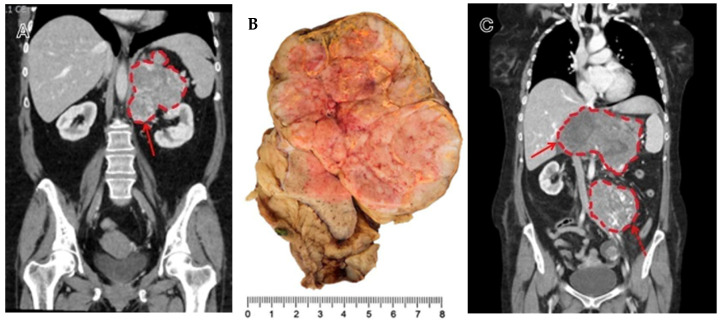
(**A**) Large, inhomogeneous adrenal tumor with areas suspected of necrosis in CT (arrow) in a 54-year-old male patient. In macro- and microscopic examination, the tumor did not show necrosis. (**B**) Metastases were detected during the course of the disease. Despite therapy with mitotane, chemotherapy, and radiotherapy, the tumor progressed. (**C**) On a CT 2 years after the primary diagnosis and 2 months before the patient’s death, a recurrent tumor in the upper abdomen with a compression of the vena portae and vena lienalis (arrow), as well as metastases in the abdominal wall next to the arteria iliaca communis (dotted arrow), were identified.

In histology, the evaluation of necrosis was sufficient for differentiating benign and malignant tumors in our study in all but two (3%) tumors. Thus, the results of our study nearly aligned with the findings presented by Walz et al. [13]. One benign adrenocortical tumor that showed necrosis in pathology in our cohort measured 83 mm and was hormonally inactive. One patient with a malignant tumor without necrosis (Figure 5) developed intraperitoneal and pulmonary metastases as well as metastases to the abdominal wall during the clinical course. The patient died 2 years after adrenalectomy. Malignant adrenocortical tumors lacking necrosis and benign tumors exhibiting necrosis have already been described [28]. Consequently, relying solely on necrosis as a predictor of malignancy in adrenocortical tumors could be misleading. Therefore, it is crucial to include other established variables, such as tumor size and endocrinological work-up, when evaluating adrenocortical tumors to increase diagnostic accuracy.

In line with previous findings, tumor diameter measured in histological specimens and tumor volume measured in cross-sectional imaging, correlated with malignancy [3]. Thus, the results of our study corroborate that the risk of malignancy increases with the size of adrenocortical tumors [14,16,27]. In a large cohort study of over 2000 patients, no adrenocortical carcinoma was found in patients with adrenal tumors measuring less than 40 mm [14]. However, the possibility of malignancy in tumors smaller than 40 mm in patients with Li–Fraumeni, Lynch, and Beckwith–Wiedemann syndromes, as well as Multiple Endocrine Neoplasia Type-1, who are at risk of developing an ACC, cannot be ruled out [29,30,31,32,33]. Thus, a thorough patient and family history must be taken, especially in young patients, as part of the evaluation of adrenocortical tumors. In patients with confirmed and suspected hereditary disease associated with adrenal disease, a prompt follow-up cross-sectional imaging control within 3 to 6 months should be performed, and an early surgical intervention should be considered regardless of the tumor size. Interestingly, the smallest malignant tumor in our patient cohort was diagnosed in a patient with Li–Fraumeni syndrome. However, owing to its small size (29 mm in diameter), it was excluded from this study.

It was previously reported that patients with cortisol- and sex hormone-secreting tumors [4,27,34] are at increased risk of malignancy. In our study, overall hormonal activity did not correlate with tumor malignancy, nor did the sole overproduction of aldosterone or cortisol alone. On the other hand, the overproduction of sex hormones highly correlated with the postoperative diagnosis of adrenocortical carcinomas. Interestingly, Walz et al. excluded aldosterone-producing adrenal tumors from their study and reported that aldosteronomas are almost always benign [13]. In our cohort, three ACCs showed overproduction of aldosterone. In contrast to two benign aldosteronomas, all of them had areas suspicious of necrosis in cross-sectional imaging and confluent necrosis in histology. Thus, the finding of necrosis facilitated the identification of rare malignant aldosteronomas in our study.

Identifying necrosis in adrenocortical tumors has several important implications for clinical practice. It reduces diagnostic ambiguity by serving as a reliable marker that, when combined with tumor size and endocrinological findings, allows for more confident detection of adrenocortical carcinoma. Identifying necrosis in preoperative imaging not only supports the decision to recommend surgical treatment but also prompts referral to a specialized center to ensure the best possible care [3]. Additionally, the presence of necrosis in histology can guide postsurgical follow-up intensity by stratifying risk, with necrosis-positive tumors requiring more frequent monitoring through imaging or endocrinological assessments to detect recurrence or metastasis early. Finally, integrating necrosis into standardized radiological and histological reporting guidelines could enhance the uniformity and reliability of evaluations, ultimately improving clinical outcomes and ensuring a higher quality of patient care.

Our study has several limitations. First, owing to the scarcity of adrenocortical carcinomas, our study cohort was small. Second, we included only adrenocortical tumors measuring at least 40 mm in size in our study. While this threshold reflects current clinical practice, as smaller adrenocortical tumors are rarely malignant and are typically not considered for surgical therapy, it introduces selection bias. This criterion may limit the applicability of our findings to smaller tumors and could affect the reproducibility of our results in broader clinical settings. Third, the study relied on retrospectively obtainable cross-sectional imaging. Both in-house and external radiological imaging were available for our patient cohort using different scanner types and non-standardized examination protocols. Importantly, when patients are referred to our clinic with external imaging, it is often not justified to repeat imaging studies solely for research purposes, particularly when the available imaging data are sufficient for clinical decision-making. However, this reliance on diverse imaging protocols can affect the consistency of necrosis assessment, as areas of necrosis may demarcate differently depending on the timing and amount of contrast application. To address these challenges, a multicentric, prospective study employing standardized examination protocols in cross-sectional imaging would enable a more comprehensive evaluation of the diagnostic accuracy of tumor necrosis in detecting malignant adrenocortical tumors within a larger patient cohort.

## 5. Conclusions

Our study confirmed that the finding of necrosis in both cross-sectional imaging and pathology serves as a widely accessible and sensitive diagnostic marker for malignancy in adrenocortical tumors. Therefore, this work-up should be established as a standard for the pre- and postoperative evaluation of all adrenocortical tumors.

## Figures and Tables

**Table 1:** Characteristics of the patients who underwent an operation for an adrenocortical tumor measuring at least 40 mm in maximum diameter.

		All Adrenocortical Tumors (*n* = 67)	Adrenocortical Adenoma (*n* = 40)	Adrenocortical Carcinoma (*n* = 27)	*p* Value
Gender ^1^	Female Male	44 (66%) 23 (34%)	27 (67.5%) 13 (32.5%)	17 (63%) 10 (37%)	0.794
Age (years) ^2^		58 (5–86)	59 (5–86)	58 (18–76)	0.720
Largest tumor diameter (mm) ^2^		60 (40–220)	50 (40–100)	100 (50–220)	<0.001
Tumor volume (cm^3^) ^2^		98.838 (2.496–4353.720)	63.901 (2.496–530.100)	378.288 (21.450–4353.720)	<0.001
Tumor side ^1^	Right Left	29 (43%) 38 (57%)	17 (42.5%) 23 (57.5%)	12 (44%) 15 (56%)	0.867
Hormonal activity ^1^	Yes No	39 (45%) 28 (55%)	21 (52.5%) 19 (47.5%)	18 (67%) 9 (33%)	0.669
Areas suspicious of necrosis in cross-sectional imaging ^1^	Yes No	32 (48%) 35 (52%)	10 (25%) 30 (75%)	22 (81%) 5 (19%)	<0.001
Areas suspicious of necrosis in histology ^1^	Yes No	27 (40%) 40 (60%)	1 (2.5%) 39 (97.5%)	26 (96%) 1 (4%)	<0.001

^1^ Count (percentage); ^2^ median (range).

**Table 2:** Overview of the availability of preoperative imaging of adrenocortical tumors.

Preoperative Cross-Sectional Imaging	All Adrenocortical Tumors (*n* = 67)	Adrenocortical Adenoma (*n* = 40)	Adrenocortical Carcinoma (*n* = 27)
CT ^1^	54 (81%)	30 (75%)	24 (89%)
MRI ^1^	26 (39%)	14 (35%)	12 (44%)
Both CT and MRI ^1^	14 (21%)	4 (10%)	10 (37%)

^1^ Count (percentage); CT—computed tomography; MRI—magnetic resonance imaging.

**Table 3:** Results of logistic regression for determining malignancy in adrenocortical tumors measuring at least 40 mm (AIC: 49266).

Coefficients	Estimate	(Standard Error)	*p*-Value
Sex	0.15327	1.01103	0.880
Age	0.04550	0.03419	0.183
Tumor diameter (mm)	0.07963	0.02533	0.002
Side	−0.06626	0.89744	0.941
Hormonal activity	1.42177	1.02056	0.164
Areas suspicious of necrosis in cross-sectional imaging	3.09420	1.06739	0.004
(Intercept)	−11.32123	3.48231	0.001

**Table 4:** Overview of hormonal overproduction in hormonally active adrenocortical tumors measuring at least 40 mm in diameter.

		All Adrenocortical Tumors (*n* = 67)	Adrenocortical Adenoma (*n* = 40)	Adrenocortical Carcinoma (*n* = 27)	*p* Value
Hyperaldosteronism ^1^	Yes No	5 (7%) 62 (93%)	2 (5%) 38 (95%)	3 (11%) 24 (89%)	0.775
Hypercortisolism ^1^	Yes No	30 (45%) 37 (55%)	19 (47.5%) 21 (52.5%)	11 (41%) 16 (59%)	0.775
Overproduction of steroid hormones ^1^	Yes No	8 (12%) 59 (88%)	0 (0%) 40 (100%)	8 (30%) 19 (70%)	<0.001
Simultaneous hypercortisolism and overproduction of steroid hormones ^1^	Yes No	6 (9%) 61 (91%)	0 (0%) 40 (100%)	6 (22%) 21 (78%)	0.014

^1^ Count (percentage).

## Data Availability

The original contributions presented in this study are included in the article.
