# Correction: Dukaczewska et al. Necrosis in Preoperative Cross-Sectional Imaging and Postoperative Histology Is a Diagnostic Marker for Malignancy of Adrenocortical Tumors. *Curr. Oncol.* 2025, *32*, 25

**DOI:** 10.3390/curroncol32040210

**Published:** 2025-04-02

**Authors:** Agata Dukaczewska, Stephan R. Marticorena Garcia, Simon Ponsel, Alexandra Webster, Frederike Butz, Eva M. Dobrindt, Johann Pratschke, Peter E. Goretzki, David Horst, Martina T. Mogl, Catarina A. Kunze

**Affiliations:** 1 Department of Surgery, Campus Charité Mitte and Campus Virchow-Klinikum, Charité–Universitätsmedizin Berlin, Corporate Member of Freie Universität Berlin, Humboldt-Universität zu Berlin and Berlin Institute of Health, Charitéplatz 1, 10117 Berlin, Germany; agata.dukaczewska@charite.de (A.D.); frederike.butz@charite.de (F.B.); eva-maria.dobrindt@charite.de (E.M.D.); johann.pratschke@charite.de (J.P.);; 2 Department of Radiology, Charité–Universitätsmedizin Berlin, Corporate Member of Freie Universität Berlin, Humboldt-Universität zu Berlin and Berlin Institute of Health, Charitéplatz 1, 10117 Berlin, Germany; stephan.marticorena-garcia@charite.de (S.R.M.G.); alexandra.webster@charite.de (A.W.); 3 Institute of Pathology, Charité–Universitätsmedizin Berlin, Corporate Member of Freie Universität Berlin, Humboldt-Universität zu Berlin and Berlin Institute of Health, Charitéplatz 1, 10117 Berlin, Germany; david.horst@charite.de (D.H.); catarina.kunze@charite.de (C.A.K.); 4 German Cancer Consortium (DKTK), Partner Site Berlin and German Cancer Research Center (DKFZ), Im Neuenheimer Feld 280, 69120 Heidelberg, Germany

Peter E. Goretzk was not included as an author in the original publication [1]. The corrected Author Contributions statement appears here. The authors state that the scientific conclusions are unaffected. This correction was approved by the Academic Editor. The original publication has also been updated.
